# Distal Reoperations after Repair of Acute Type A Aortic Dissection—Incidence, Causes and Outcomes

**DOI:** 10.31083/j.rcm2307228

**Published:** 2022-06-24

**Authors:** Igor Vendramin, Daniela Piani, Andrea Lechiancole, Sandro Sponga, Daniele Muser, Massimo Imazio, Francesco Onorati, Elisabetta Auci, Uberto Bortolotti, Ugolino Livi

**Affiliations:** ^1^Azienda Sanitaria Universitaria Friuli Centrale, Cardiothoracic Department, Via Pozzuolo 11, 33100 Udine, Italy; ^2^Azienda Ospedaliero-Universitaria di Verona, Cardiothoracic and Vascular Department, 37126 Verona, Italy; ^3^Department of Medical Area (DAME), University of Udine, 33100 Udine, Italy

**Keywords:** aortic dissection, redo operations, reoperations, arch replacement

## Abstract

**Background and Aim of the Study:**

In patients with acute type A aortic dissection (A-AAD) whether initial repair should include also aortic arch replacement is still debated. We aimed to assess if extensive aortic repair prevents from reoperations patients with A-AAD.

**Methods:**

Outcomes after distal reoperation following repair of A-AAD (n = 285; 1977 to 2018) were analysed in 22 of 226 who underwent ascending aorta/hemiarch replacement (Group $1^{\text{R}}$) and 7 of 59 who had ascending aorta/arch replacement (Group $2^{\text{R}}$).

**Results:**

Distal reoperation was more common in Group $1^{\text{R}}$ (n = 22) than in Group $2^{\text{R}}$ (n = 0) (*p *< 0.001) while thoracic endovascular stenting was more frequent in Group $2^{\text{R}}$ (7 vs 3, *p *< 0.001). Indications for reoperation were pseudoaneurysm at distal anastomosis (n = 4, 18%) and progression of aortic dissection (n = 18, 82%) in Group $1^{\text{R}}$. Indication for thoracic endovascular stenting was progressive aortic dissection in 3 patients of Group $1^{\text{R}}$ and in 6 of Group $2^{\text{R}}$. Second reoperation was required in 2 patients from Group $1^{\text{R}}$ (2%) during a mean follow-up of 5 years. Median follow-up was 4 years in Group $1^{\text{R}}$ and 7 years in Group $2^{\text{R}}$ (*p* = 0.36). Hospital mortality was 14% in Group $1^{\text{R}}$ and 0% in Group $2^{\text{R}}$ (*p* = 0.3). Actuarial survival is 68 ± 10%, and 62 ± 11% for Group $1^{\text{R}}$ and 100% for Group $2^{\text{R}}$ at 5 and 10 years (*p* = 0.076).

**Conclusions:**

Distal reoperations after A-AAD repair have an acceptable mortality. An extensive initial repair has lower rate of reoperation and better mid-term survival and should be indicated especially for young patients in experienced centers.

## 1. Introduction

Acute type A aortic dissection (A-AAD) is a life-threatening condition where surgery is aimed to prevent death from aortic rupture, malperfusion and severe aortic valve regurgitation [1, 2]. Regardless of the surgical techniques employed and the extension of the repair, part of the diseased aortic tissue is left in place and this may become the source of late complications [3, 4]. The tract most prone to develop post-repair sequelae is represented by the aortic arch, when it is not replaced during initial operation, or the distal aorta when the false lumen remains patent [5]. Other adverse events include false aneurysms formation at the suture lines and dilatation of the aortic root with onset or progression of aortic valve incompetence [6].

Although supracommissural replacement of the ascending aorta may be the simplest method to achieve a successful, low-risk repair in most patients with A-AAD, this limited approach may predispose to the need for subsequent distal reoperations [4, 7, 8]. Reoperations in patients after repair of acute A-AAD are challenging procedures particularly when total arch replacement is required. Therefore, in patients with A-AAD there is a current trend to support total arch replacement as index operation rather than a less complex procedure, although this issue is still not completely solved [7, 9, 10].

The aim of this study was to investigate the incidence, causes and outcomes of distal reoperations following repair of A-AAD and to analyse long-term outcomes in a series of patients from a single institution experience.

## 2. Material and Methods

We have reviewed patients who underwent repair of A-AAD, from 1977 to 2018, at our Institution. All patients discharged were analysed retrospectively to assess incidence and causes of late distal reoperations, which included either surgical procedures or thoracic endovascular aortic repair (TEVAR). The primary end-points of the study were analysis of mortality and assessment of late outcomes. The study was conducted in accordance with the Declaration of Helsinki, and the protocol was approved by the Institutional Review Board (approval number: 013/2020_IRB Tit. III cl.32 fasc.32), without the need for patient informed consent.

*Patient population*: A-AAD repair was performed by supracommissural graft replacement of the ascending aorta with hemiarch (Group 1, 81%) and total arch (Group 2, 19%) extension. At initial repair, hospital mortality was 21% in Group 1 and 14% in Group 2. Overall mortality improved during the study period from 34% (1977–1994) to 22% (1995–2009) and 12% (2010–2018) for Group 1 and from 38% (1995–2009) to 8% (2010–2018) for Group 2.

A total of 285 hospital survivors were available for inclusion and analysis, 226 in Group 1 (79%) and 59 in Group 2 (21%). In Group 1 mean age was 64 ± 13 years (range, 20 to 81 years), 72% were males, 7% had a bicuspid aortic valve and 2% Marfan’s syndrome. In Group 2 mean age was 61 ± 11 years (range, 20 to 81 years), 75% were males and 3% had Marfan’s syndrome. Other main clinical characteristics and surgical data of both Groups are summarized in Tables 1,2. Mean follow-up in the entire population was 5 ± 1 years (range, 2 months to 17 years). Median follow-up was 6.7 years for Group 1 patients (range,1 month to 42 years), and 4 years for Group 2 patients (range, 4 months to 23 years).

**Table 1.** **Baseline characteristics of survivors of acute type A dissection**.

	Group 1 (n = 226)	Group 2 (n = 59)	*p* value
Clinical profile			
Male sex, n (%)	163 (72)	44 (75)	0.71
Median age (years)	64 (18–86)	64 (18–86)	0.144
	n. (%)	n. (%)	
Chronic renal failure	7 (3)	5 (8)	0.08
COPD	17 (8)	5 (8)	0.87
Marfan’s syndrome	5 (2)	2 (3)	0.64
Preoperative anticoagulation	14 (6)	6 (10)	0.34
Previous cardiac surgery	13 (6)	2 (3)	0.45
Bicuspid aortic valve	16 (7)	-	0.03
CAD	21 (10)	4 (7)	0.47
Chronic AF			
Presentation			
Thoracic pain	131 (79)	44 (81)	0.74
Tamponade/shock/hypotension	56 (33)	17 (30)	0.68
Syncope	32 (19)	12 (22)	0.65
Neurologic damage	24 (14)	13 (24)	0.11
Coma	1 (1)	3 (6)	0.02
Acute renal failure	13 (12)	7 (14)	0.73
Risk factors			
Hypertension	156 (71)	48 (81)	0.11
Smoke	47 (22)	16 (27)	0.38
Dyslipidemia	25 (11)	7 (12)	0.94
Diabetes	7 (3)	3 (5)	0.50

COPD, Chronic obstructive pulmonary disease; CAD, Coronary artery disease; AF, Atrial fibrillation.

**Table 2.** **Surgical data of survivors after repair of type A acute aortic dissection**.

	Group 1 (n =226)	Group 2 (n = 59)	
Surgical procedures			
Sopracommissural AA replacement	181		
- with AV repair	12		
- with AV replacement	10		
- with CABG	3		
- with MV replacement	1		
- with AV repair, CABG	1		
Bentall/Cabrol	41		
- with CABG	3		
T. David	2		
Yacoub	1		
T. David, CABG	1		
Sopracommissural AA replacement		22	
- with arch replacement		20	
- with arch replacement, Bentall		1	
- with arch replacement, CABG		1	
Sopracommissural AA replacement		30	
- with classic ET,		25	
- classic ET, T. David		2	
- classic ET, Bentall		1	
- classic ET, AV repair		1	
- classic ET, AV replacement		1	
Sopracommissural AA replacement		7	
- with frozen ET		5	
- with frozen ET, T. David		2	
			*p* value
Mean CPB time, min	215 ± 73	257 ± 84	<0.001
Mean ACC time, min	125 ± 63	155 ± 70	0.003

AA, Ascending aorta; AV, Aortic valve; CABG, Coronary artery bypass grafting; MV, Mitral valve; ET, Elephant trunk; CPB, Cardiopulmonary bypass; ACC, Aortic cross-clamp.

*Patient follow-up*: All discharged patients were entered in a follow-up program including periodical visits by a dedicated team, 1 and 6 months after surgery and on a yearly basis thereafter. Transthoracic 2D echocardiograms were used to assess cardiac function and stability of the repair calculating aortic root and arch diameters and evidencing presence of residual intimal flaps. Angio-computed tomography (CT) was generally performed at 1, 6 and 12 months postoperatively and repeated whenever considered indicated. All data were registered in a specific database and used for analysis and comparison with those collected preoperatively. Information on patients was also obtained from phone interviews or contact with relatives or family physicians.

*Surgical technique*: All reoperations were performed through a repeat median sternotomy. Cannulation for cardiopulmonary bypass (CPB) was generally through the right axillary artery and the right atrium or a femoral vein. Under moderate hypothermia (24–26 °C) the ascending aorta graft was clamped, opened at the previous distal suture line and sutures, felts and other debris removed. Selective antegrade cerebral perfusion was generally obtained through the right axillary artery and by direct cannulation of the left carotid artery. The epiaortic vessels were then detached from the arch which was excised at the origin of the left subclavian artery. The distal aortic stump was prepared with biological glue and sandwiched with strips of Teflon. Reconstruction of the aortic arch was obtained mainly with frozen elephant trunk (ET) procedure using a quadrifurcated graft. During the distal suture a tip-cut Foley catheter was inflated into the graft and used for splanchnic perfusion which was subsequently obtained through the lateral branch of the graft. All other anastomoses and surgical procedures on the aortic root or valve were performed during rewarming.

*Statistical analysis*: Continuous variables, expressed as means ± standard deviations if normally distributed or medians (minimum - maximum range) if not, were tested for normal distribution using the 1-sample Kolmogorov–Smirnov test. Continuous variables were compared using independent sample parametric (unpaired Student t) or non-parametric (Mann-Whitney U) tests. Categorical data, expressed as counts and percentages, were compared using Chi-Square or Fisher Exact test when appropriate. Survival curves were generated by Kaplan–Meier method and compared by log rank test. A competing risk analysis, based on the Fine and Gray risk time-to-event model, was used for analysis of time to reintervention [11]. The cumulative incidence function was employed for estimation of incidence of outcomes while taking competing risk into account. Comparison between curves was made by the Gray’s test [12], while event times were measured from the date of surgery. Univariable and multivariable Cox proportional hazards analyses were used to test the association between time to reintervention and baseline covariates. Event times were measured from the date of surgery. All potential confounders were initially entered into the multivariable model on the basis of known clinical relevance; then a model reduction was performed by excluding variables with a *p*-value > 0.20 based on the log-likelihood test. The proportional hazards assumption was assessed globally and for all variables using Schoenfeld’s residuals test. Two-tailed tests were considered statistically significant at 0.05 level. Statistical analysis was performed using IBM-SPSS software Version 22.0 (IBM Corp., Armonk, NY, USA) and in R version 4.0.3 software (R Foundation for Statistical Computing, Vienna, Austria).

## 3. Results 

*Patient profile*: Distal reoperation was required in 29 patients, in 22 patients of Group 1 (Group $1^{\text{R}}$; 9.7%) and in 7 of Group 2 (Group $2^{\text{R}}$, 12%) and were performed from 2002 and 2019. In Group $1^{\text{R}}$ reoperation was performed at a median distance of 4 years from initial repair (range, 2 months to 20 years) and in Group $2^{\text{R}}$ after a median interval of 4 months (range, 1 month to 2 years). Mean age at reoperation in Group $1^{\text{R}}$ was 63 ± 14 years, 82% of patients were males and 4.5% had Marfan’s syndrome; in Group 2$2^{\text{R}}$ mean age at reoperation was 62 ± 3 years, 71% of patients were males while none had Marfan’s syndrome. Other characteristics of patients undergoing distal reoperation are summarized in Table 3.

**Table 3.** **Clinical characteristics of patients undergoing distal reoperation**.

	Group $1^{\text{R}}$ (n = 22)	Group $2^{\text{R}}$ (n = 7)	*p* value
Clinical profile			
Male sex, n (%)	18 (82)	5 (71)	0.46
Mean age at 1st operation, years (range)	57 ± 13 (18–79)	61 ± 4 (58–66)	0.22
Mean age at reoperation, years (range)	63 ± 14 (20–81)	62 ± 3 (58–66)	1
Chronic renal failure, n (%)	1 (4.5)	-	0.76
Mean preoperative creatinine (µmol/L)	1.16 ± 0.3 (0.72–2.12)	1.18 ± 0.41 (0.74–1.86)	0.9
COPD, n (%)	4 (18)	2 (28)	0.46
Marfan’s syndrome, n (%)	1 (4.5)	-	0.76
Preoperative anticoagulation, n (%)	8 (36)	1 (14)	0.27
CAD, n (%)	-	2 (28)	0.052
Preoperative AF, n (%)	1 (4.5)	1 (14)	0.43
Risk factors			
Hypertension, n (%)	15 (68)	6 (86)	0.35
Smoke, n (%)	6 (27)	3 (43)	0.37
Dyslipidemia, n (%)	2 (9)	-	0.57
Diabetes, n (%)	1 (4.5)	-	0.76

COPD, Chronic obstructive pulmonary disease; CAD, Coronary artery disease; AF, Atrial fibrillation.

*Surgical data*: Causes of reintervention and surgical details are summarized in Table 4.

**Table 4.** **Surgical data of patients requiring reoperation**.

	Group $1^{\text{R}}$ (n = 22)	Group $2^{\text{R}}$ (n = 7)	*p* value
*Indication for reoperation*			
	n (%)	n (%)	
Distal false lumen dilatation	18 (82)	6 (86)	0.69
with proximal false aneurysm	4	-	
Distal false aneurysm	4 (18)	1 (14)	0.65
*Surgical procedures*			
Frozen ET procedure	10	-	
with MBP	2		
with NCS replacement	1		
Aortic arch replacement	4	-	
with MPB	1		
with MVR + TVR	1		
Classic ET procedure	2		
with MBP	1		
Distal suture reinforcement	2	-	
Replacement of proximal descending aorta	1	-	
TEVAR	3 (14)	7 (100)	<0.001
*Intraoperative details*			
Median CPB time, min (range)	232 (125–453)	-	
Median aortic cross-clamp time, min (range)	125 (31–268)	-	
Median circulatory arrest time, min (range)	49 (15–108)	-	

ET, Elephant trunk; MBP, Modified Bentall procedure; NCS, Non-coronary sinus; MVR, Mitral valve repair; TVR, Tricuspid valve repair; TEVAR, Thoracic endovascular aortic repair; CPB, Cardiopulmonary bypass.

In Group $1^{\text{R}}$ indication for reoperation was progressive enlargement of the false lumen in 18 patients (82%), associated to pseudoaneurysm formation at the proximal aortic graft anastomosis in 4, and false aneurysm at the distal suture line in 4 (18%). Main surgical procedures were: arch replacement with frozen ET in 10 (45%), isolated arch replacement in 4 (18%), arch replacement with standard ET in 2 (9%), distal suture reinforcement in 2 (9%) and replacement of proximal descending aorta in 1 (5%); 3 patients underwent TEVAR. In Group $2^{R}$distal false lumen dilatation occurred in 6 patients (86%) and distal pseudoaneurysm formation in 1 (14%); all underwent TEVAR. Associated procedures were performed in 6 patients of Group $1^{\text{R}}$: a modified Bentall procedure in 4, mitral and tricuspid valve repair in 1 and replacement of the non-coronary sinus of the aortic valve in 1.

*Early and late results*: There were 3 in-hospital deaths (10%), all of them occurring in Group $1^{\text{R}}$ patients (14%) (Table 5). Causes of death were aortic-related (aortic rupture) in 2 patients and sepsis in 1. Median follow-up after reoperation is 3.9 years (range, 2 months to 18 years) for Group $1^{\text{R}}$ and 6.8 years (range, 2 to 11 years) for Group $2^{\text{R}}$ patients (*p* = 0.04).

**Table 5.** **Early results at reoperation**.

	Group $1^{\text{R}}$ (n = 22)	Group $2^{\text{R}}$ (n = 7)	*p* value
30-day mortality, n (%)	3 (14)	-	0.30
Aortic-related, n (%)	2 (67)		
Sepsis, n (%)	1 (33)		
Chest re-exploration, n (%)	1 (4.5)	-	0.76
Acute renal failure, n (%)	8 (36)	-	0.075
Dialysis, n (%)	2 (9)	-	0.57
Cerebral ischemia, n (%)	2 (9)	-	0.57
Mechanical ventilation ≥72 h, n (%)	5 (23)	-	0.22
Median ICU stay, days (range)	4 (1–40)	1 (1–3)	0.06
Median hospital stay, days (range)	20 (7–81)	9 (3–15)	0.006

ICU, Intensive care unit.

In Group $1^{\text{R}}$ there were 4 late deaths, all due to aortic-related events (aortic rupture in 3 and aortic thrombosis in 1). A second reoperation was required in 2 patients of Group $1^{\text{R}}$ who required TEVAR and proximal reoperation, respectively. After multivariable adjustment, only age (HR 0.98, 95% CI 0.77–3.01, *p* = 0.03) resulted as an independent predictor of reintervention (Table 6).

**Table 6.** **Univariable and multivariable Cox proportional hazards analysis of baseline covariates in relation to distal reintervention**.

	Reintervention			
	Univariable		Multivariable	
	HR (95% CI)	*p* value	HR (95% CI)	*p* value
Age	0.97 (0.95–1.03)	0.09	0.98 (0.77–3.01)	0.03
Male gender	1.31 (0.53–3.22)	0.56		
Hypertension	1.17 (0.52–2.65)	0.71		
Smoking habit	1.19 (0.51–2.79)	0.69		
Dislipidemia	3.36 (0.46–5.72)	0.23		
Diabetes	2.30 (0.01–15.00)	0.60		
Chronic kidney disease	1.05 (0.01–3.90)	0.54		
Atrial fibrillation	1.19 (0.28–5.04)	0.81		
COPD	3.02 (0.70–7.97)	0.55		
Stroke	0.04 (0.01–3.10)	0.43		
LVEF	0.97 (0.94–1.08)	0.72		
Marfan syndrome	1.14 (0.15–8.46)	0.89		
Previous cardiac surgery	0.81 (0.19–3.41)	0.77		
Bicuspid aortic valve	2.83 (1.06–7.55)	0.03	2.96 (0.99–8.38)	0.14
Moderate to severe AR	2.01 (0.92–4.39)	0.08	1.79 (0.81–3.93)	0.15
AA vs arch replacement	0.58 (0.24–1.39)	0.22		
AA diameter	0.90 (0.72–1.14)	0.41		
Arch diameter	0.55 (0.14–2.21)	0.40		
Aortic valve replacement	1.21 (0.17–8.94)	0.84		
CPB time	1.01 (0.98–1.03)	0.31		
Circulatory arrest time	1.00 (0.98–1.01)	0.67		

COPD, Chronic obstructive pulmonary disease; LVEF, Left ventricular ejection fraction; AR, Aortic regurgitation; AA, Ascending aorta; CPB, Cardiopulmonary bypass.

**Fig. 1.**
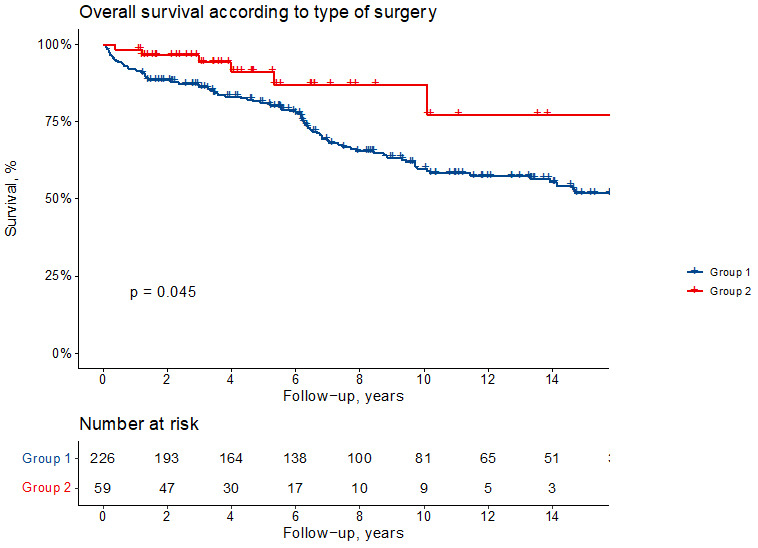
**Kaplan-Meier survival curves showing overall survival according to the type of surgical repair**. Group 1 indicates patients with ascending aorta and hemiarch replacement and Group 2 those with arch replacement.

Survival at 5, 10 and 15 years, according to Kaplan-Meier estimates, is 92 ± 2% vs 98 ± 2, 81 ± 3% vs 91 ± 4%, 60 ± 4% vs 87 ± 6% for Group 1 and 2, respectively (log-rank *p* = 0.049) (Fig. 1).

**Fig. 2.**
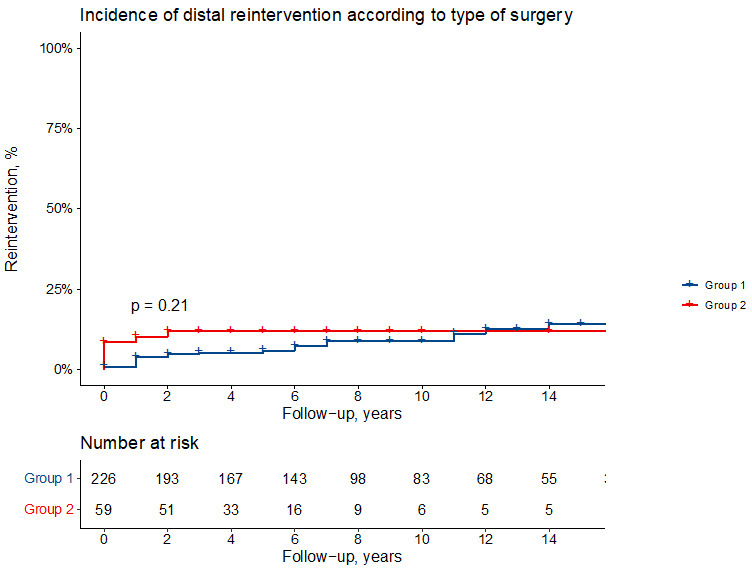
**Cumulative incidence function showing the incidence of distal reintervention (death as competing risk) in Group 1 and Group 2 patients**.

Freedom from distal reoperation at 5, 10 and 15 years, according to cumulative incidence functions, is 99 ± 1% vs 91 ± 4%, 94 ± 1% vs 87 ± 5% and 90 ± 2% vs 87 ± 5% in Group 1 and 2, respectively (log-rank *p* = 0.18) (Fig. 2).

**Fig. 3.**
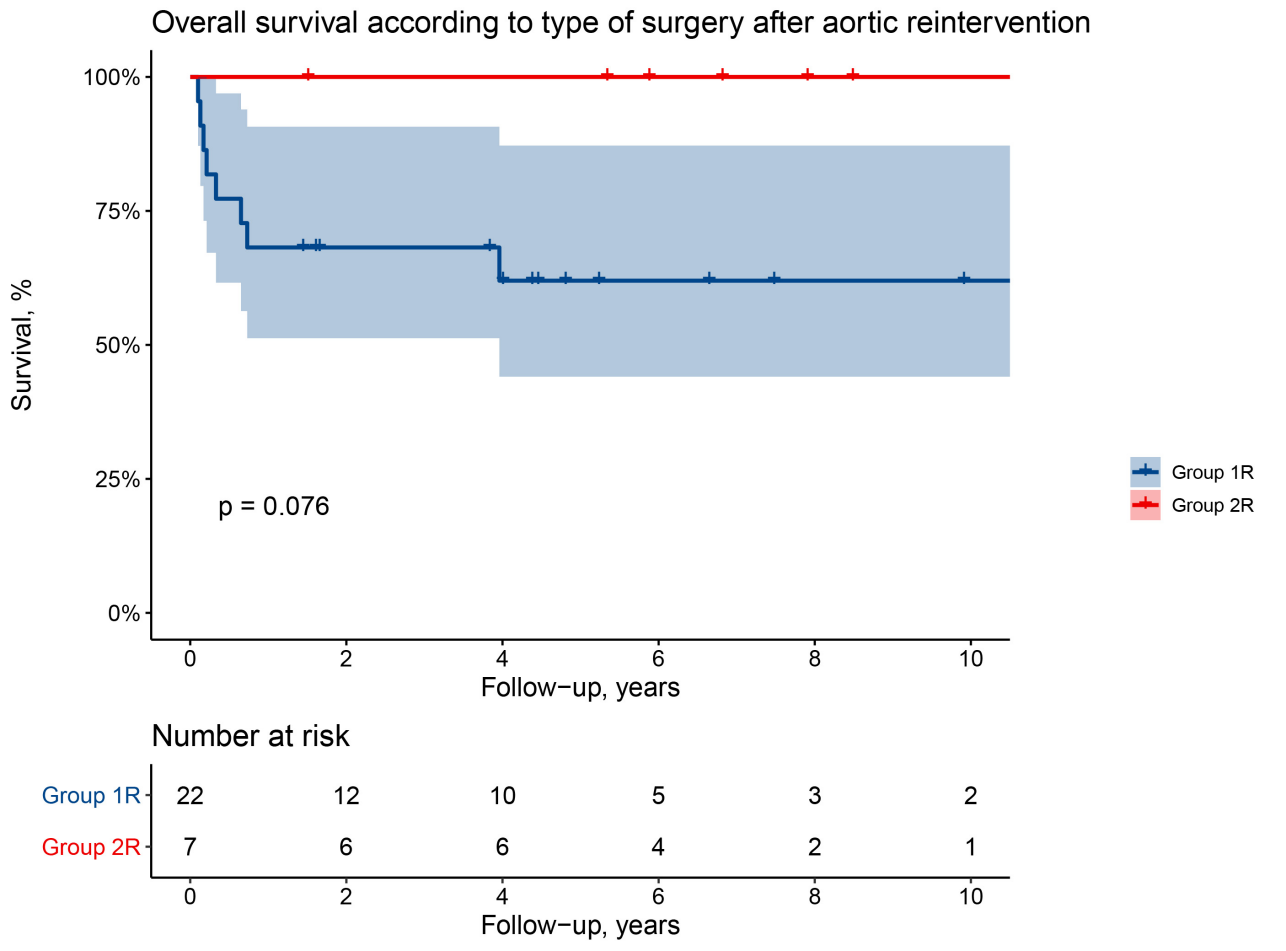
**Overall survival after aortic reintervention (surgery or thoracic endovascular aortic repair)**.

Survival according to Kaplan-Meier estimates in patients of Group $1^{\text{R}}$ at 1 is 68 ± 10% and 62 ± 11% at 5 and 10 years while it is 100% at every interval for Group $2^{\text{R}}$ (log-rank *p* = 0.076) (Fig. 3).

## 4. Discussion

Limited replacement of the ascending aorta with possible hemiarch extension is a reasonable approach for patients during repair of A-AAD, also when the aortic arch is involved by dissection, in the absence of arch dilatation, intimal arch tears or malperfusion of the cerebral vessels [7]. However, in the presence of significant dilatation or extensive aortic arch involvement by dissection with intimal tears, arch replacement becomes mandatory [9].

Survivors of A-AAD repair are at continuous risk of developing late complications, particularly related to expansion of the distal false lumen, progression of dissection or pseudoaneurysm formation [12]; moreover, persistence of residual false lumen has been recognized also as an independent risk factor for poor long-term survival [5, 13, 14, 15]. Therefore, the possibility of an unfavourable evolution of the underlying disease should not be overlooked when selecting the best surgical option even if almost always decision is required in an emergency situation. Compared to patients who had immediate total arch replacement the incidence of distal reoperations is higher in those with limited supracommissural replacement of the ascending aorta; despite most reoperations being performed in an elective setting, the need for arch replacement at a later stage may represent an important surgical challenge and a significant burden for the patient [3].

Few studies have examined the results of reoperation after A-AAD repair, especially focusing on distal aortic reoperation and the long-term outcome of these patients [8]. Most of them compare extensive and limited index arch repair showing conflicting results and recommendations, likely due to heterogeneous patient populations, surgical experience and short follow-up.

From the available data it is still uncertain whether total arch replacement should be extended to most patients as initial approach in A-AAD repair, particularly since the immediate higher technical complexity and increased operative risks must be weighed against those of a fastest operation with lower risks. However, should sufficient evidence be available indicating that arch replacement is associated to a lower risk of distal reoperations, better late survival and even acceptable mortality at possible reoperations, arch replacement as first-step repair in patient with A-AAD would be strongly supported especially in younger patients.

The purpose of the present study was to investigate and compare two subsets of patients, followed for over 20 years, after A-AAD repair using either a limited or a more radical surgical approach, to verify the incidence and results of distal reoperations from a single center experience. In the initial series we observed an evident improvement of the early results with a hospital mortality which dropped, in the study period, from 34% to 12% and from 38% to 8% in patients with limited ascending aorta and hemiarch replacement and those with total arch replacement, respectively. Operative mortality for A-AAD is reported by others to be still quite high, although it has definitely improved in recent years and this has been confirmed also by our experience [3, 16, 17, 18, 19, 20].

The results of our study indicate that despite a substantial follow-up length the overall number of reoperations was limited regardless the technique employed at index operation.

Out of 226 survivors with limited repair, including however hemiarch replacement, less than 10% required reoperation at a median distance of 4 years while 12% of those having total arch replacement underwent a much earlier reoperation (median interval of 4 months).

Our data are confirmed also by the multicenter report by Pan *et al*. [21] who, in a series of 1159 patients at a maximum follow-up of 10 years, observed a low reoperation rate; interestingly, incidence of reoperations was not influenced by the extent of initial repair.

The cause of reoperation was in most cases distal progression of false lumen dilatation despite almost 60% of patients having a classic (n = 30) or frozen (n = 7) ET in the arch replacement group. This indicates that most likely the classic ET technique, as described originally [22], does not provide adequate support to the distal aorta as well as any favourable effect on reduction of false lumen patency; indeed, in patients undergoing reoperation a more extensive use of the frozen ET has been performed with no further need for distal reinterventions after a median interval from 4 to 7 years, except in one patient who required TEVAR.

Most of the patients in the hemiarch replacement group required an open reoperation with acceptable mortality and mid-term results. Although they were younger at first operation compared to the uncomplicated patients of the same group, all in-hospital and 4 late deaths were due to aortic-related events, and a second reoperation was required in 2 patients due to aortic complications. This scenario suggests both a more aggressive initial aortic pathology and a more challenging management of residual dissection of the arch and the descending aorta.

Conversely, all patients in the arch replacement group requiring a second reoperation underwent TEVAR without in-hospital mortality and an actuarial survival of 100% at every interval, indicating that the treatment of residual dissected aorta after arch replacement may be performed easily with excellent early and late results. Similar results in late reoperations have been reported by others; however, since most studies include also proximal repeat procedures, the real impact of distal reoperations on survival is not always clear [3, 4, 12, 15, 18].

The results of our study may be summarized as follows: (1) regardless of the initial technique of repair of A-AAD, the incidence of late reoperations is low; (2) patients who had hemiarch replacement at index operation have a lower late survival compared to those undergoing immediate arch replacement; (3) when initial A-AAD repair is limited to the ascending aorta or even extended to the hemiarch, reoperation requires open arch replacement, while after initial arch replacement TEVAR alone is effective to treat subsequent distal complications; (4) TEVAR may be effectively performed with 0% mortality, even for unusual postoperative complications [23], while elective reoperation with arch replacement has an acceptable risk.

The major limitation of the paper may be represented by the small sample of patients requiring reoperation during a long follow-up period. On the other hand, this could indicate the effectiveness of surgical procedures employed at index operation when dealing with an often complex disease, extremely fragile tissues and the need for extended repair. Furthermore, the real number of patients requiring reoperation might be underestimated if we should add the few cases of late aortic-related deaths in patients in whom long-term complications were probably misdiagnosed or who were not referred in time for reoperation; despite this the overall need for late reoperation remains substantially small due to the thorough follow-up evaluation performed in our center in patients after A-AAD repair. Finally, the aim of this study was not to compare limited ascending aorta versus extensive arch repair as initial management for A-AAD but rather to analyze the safety of late reoperation in a referral center to help in decision-making at first operation, especially for patients undergoing index repair in lower volume centers and for surgeons with limited experience in aortic surgery.

## 5. Conclusions

Our data support a conservative approach at time of repair of A-AAD in most patients and therefore limited replacement of the ascending aorta or hemiarch can be considered a sound approach to A-AAD repair. Late complications which may ensue after A-AAD repair can be corrected electively and with low risks. A more extensive arch repair as index operation should be reserved especially to young subjects with A-AAD in experienced aortic referral centers, since this approach seems to be protective from potential unfavourable evolutions of the residual aortic dissection.
